# Conjunctival administration of H38Δ*wbkF* rough vaccine as an effective strategy to protect against *Brucella ovis* infection while minimizing serological interference

**DOI:** 10.1186/s13567-025-01693-8

**Published:** 2026-03-08

**Authors:** Nerea Lopez, Sara Andrés-Barranco, M. Jesús De Miguel, Amaia Zúñiga-Ripa, Aitor Elizalde-Bielsa, Miriam Salvador-Bescós, Maite Iriarte, Montserrat Barberán, José María Blasco, Ignacio Moriyón, Raquel Conde-Álvarez, Pilar M. Muñoz

**Affiliations:** 1 Department of Microbiology and Parasitology, Instituto de Investigación Sanitaria de Navarra (IdiSNA), University of Navarra, 31008 Pamplona, Spain; 2 Department of Animal Science, Centro de Investigación y Tecnología Agroalimentaria de Aragón (CITA), Av. Montañana, 930, 50059 Zaragoza, Spain; 3 Instituto Agroalimentario de Aragon-IA2, CITA-Universidad de Zaragoza, 50009 Zaragoza, Spain; 4 Unité de Recherche en Biologie Des Microorganismes (URBM), Department of Biology, Namur Research Institute for Life Sciences (NARILIS), University of Namur, Namur, Belgium; 5 Departament of Animal Pathology, University of Zaragoza, Zaragoza, Spain

**Keywords:** *Brucella*, sheep, conjunctival administration, vaccine, serological test, ovine brucellosis

## Abstract

Sheep brucellosis is a reproductive disease caused by *Brucella melitensis* and *B. ovis*, Gram-negative bacteria that differ in surface antigens: *B. melitensis* carries a smooth (S) lipopolysaccharide (LPS) with a characteristic O-polysaccharide (O-PS), while *B. ovis* bears a rough (R) LPS lacking O-PS. Only the former is zoonotic and of public health concern, but both cause significant economic losses, thus requiring effective vaccines. The sole vaccine available, *B. melitensis* Rev1, protects against both infections but induces antibodies detected by S-LPS-based tests used for *B. melitensis* surveillance. Since controlling this zoonotic species is a priority, Rev1 is banned in *B. melitensis*-free areas. Consequently, *B. ovis* (detected by R-LPS-based tests) remains endemic or re-emerges, and an R brucellosis vaccine is needed. Previously, we demonstrated that subcutaneous vaccination with *B. melitensis* R mutant H38Δ*wbkF* protects rams against *B. ovis* similarly to Rev1, without interfering in the official S-LPS-based Rose Bengal and Complement Fixation tests. However, H38Δ*wbkF* elicits R-LPS antibodies that interfere with *B. ovis* tests. Here, we explored two strategies to minimize this interference: (i) altering H38Δ*wbkF* relevant diagnostic epitopes by constructing *wadB* and *wadC* mutants deleted in sugars of the R-LPS core tetrasaccharide branch; and (ii) administering H38Δ*wbkF* conjunctivally, a route known to reduce antibody responses to S brucellosis vaccines. While the core-defective mutants were over-attenuated and failed to protect mice, the conjunctival route preserved H38Δ*wbkF* efficacy in rams and significantly reduced serological interference. Conjunctival H38Δ*wbkF* vaccine is thus a suitable tool for *B. ovis* eradication in *B. melitensis*-free areas.

**Supplementary Information:**

The online version contains supplementary material available at 10.1186/s13567-025-01693-8.

## Introduction

*Brucella* is a genus of Gram-negative facultative intracellular bacteria with a worldwide distribution that includes zoonotic and non-zoonotic species and biovars. Most *Brucella* species have a preferential host, encompassing ruminants, suids, canids, camelids, marine mammals, and various wildlife species [1]. Ovine brucellosis is caused by either *B. melitensis*, a highly zoonotic agent, or the non-zoonotic *B. ovis*. Both species produce similar clinical manifestations, including genital lesions, increased perinatal mortality, abortions, and infertility [2–4]. While *B. melitensis* exhibits a smooth (S) lipopolysaccharide (LPS) with an *N*-formyl-perosamine O-polysaccharide (O-PS) crucial for virulence, *B. ovis* lacks this O-PS and therefore presents a rough (R) LPS [1]. Although both bacteria share the R-LPS structure, this difference significantly impacts host–pathogen interactions and serodiagnostic tests. Whereas *B. melitensis* serodiagnosis requires S-LPS-based tests [5, 6], hot saline extracts (HS) rich in R-LPS and outer membrane proteins are the most effective antigen for *B. ovis* serodiagnosis [5, 7–9].

As *B. melitensis* is the primary cause of human brucellosis worldwide, efforts to control or eradicate ovine brucellosis are mostly focused on this species [10–13]. In several developed countries, *B. melitensis* eradication has been achieved through long-term government investment in vaccination and strict test-and-slaughter policies. The live attenuated *B. melitensis* Rev1 vaccine [14], the only option for small ruminants, has been instrumental in most cases. The strategy commonly used for eradication consists of vaccinating young replacements and serological surveillance in adults combined with test-and-slaughter. Rev1 protects against both *B. melitensis* and *B. ovis* [15–17] and is safe in rams [18]. However, Rev1 induces an antibody response that interferes in S-LPS-based diagnostics such as the official Rose Bengal (RBT) and Complement Fixation (CFT) tests or S-LPS indirect enzyme-linked immunosorbent assay (iELISA) [6]. Moreover, Rev1 remains virulent for humans and is resistant to streptomycin, a first-line antibiotic used in human brucellosis [19]. These drawbacks have led to the ban of Rev1 in regions where *B. melitensis* has been eradicated, resulting in the reemergence of *B. ovis* in unprotected areas [3, 20, 21]. Also, *B. ovis* is endemic in regions where Rev1 vaccination was never implemented [21, 22]. These challenges underscore the urgent need for a vaccine against *B. ovis* that does not interfere with *B. melitensis* serosurveillance and, ideally, that has minimal impact on *B. ovis* diagnostics, thus enabling the implementation of complementary test-and-slaughter measures where feasible.

Efforts to develop *B. ovis* vaccines have been primarily based on inactivated or subcellular formulations combined with novel adjuvants, and some have shown promising results in rams [23–27]. However, none has been evaluated under field conditions or advanced toward commercialization probably because vaccines that require costly adjuvants or repeated doses are economically unfeasible or impractical for the sheep farming sector. Overall, live attenuated vaccines remain the most cost-effective strategy for brucellosis vaccination [28, 29]. *B. abortus* RB51, an R spontaneous mutant currently marketed as cattle vaccine, yields unsatisfactory results in rams [30]. A *B. ovis* mutant in a putative ABC transporter, encapsulated in alginate, was reported to be protective in rams [31], but methodological flaws raise concerns about this study [32]. More recently, a Rev1Δ*wzm* mutant defective in the O-PS translocation system was shown to be protective against *B. ovis* in rams [33]. However, *wzm* mutants trigger antibodies that interfere in RBT and CFT for at least 4–6 months after vaccination [34–37] and, when constructed in a Rev1 background, retain streptomycin resistance.

In a previous study, our group developed several genetically engineered live vaccine candidates using different attenuation and tagging strategies and evaluated their efficacy in rams [32]. Among them, a mutant deleted in *wbkF*, the gene encoding the undecaprenyl-glycosyltransferase necessary for initiating O-PS polymerization, showed protection similar to that of Rev1 and did not interfere in RBT or CFT. However, it triggered antibodies interfering in the World Organisation for Animal Health (WOAH)-recommended [38] *B. ovis* agar gel immunodiffusion test (AGID) and iELISA with the HS antigen for 5 or more than 7 months, respectively [32]. In the same study, a CO_2_-independent *B. ovis* construct (Bov:CA) [39] mutated in the LPS core glycosyltransferase WadB (Bov:CAΔ*wadB*) failed to confer protection in rams. Interestingly, Bov:CAΔ*wadB* hardly interfered in standard AGID whereas it did interfere in AGID performed with the HS Δ*wadB* homolog [32]. Since R-LPS is a major component of HS (see above), these observations suggest that modifying the R-LPS epitopes could mitigate the serological interference of *B. ovis* R vaccines constructed in a protective background.

In the current study, we investigated two strategies to improve H38Δ*wbkF* by reducing the interference in *B. ovis* serological diagnostics. First, we explored whether introducing defects into the LPS core lateral branch of H38Δ*wbkF* could hinder the recognition of vaccine-induced antibodies in *B. ovis* standard serological tests, thereby obtaining a vaccine enabling the differentiation of infected and vaccinated animals (DIVA). To this end, we constructed two new mutants (H38Δ*wbkF*Δ*wadB* and H38Δ*wbkF*Δ*wadC*) and assessed them in laboratory models. Also, since the conjunctival route is known to reduce the intensity and persistence of Rev1-derived antibodies [40–42], we evaluated the protection and serological response of H38Δ*wbkF* administered conjunctivally to rams using a commercial conjunctival Rev1 (Ocurev^®^) as a protective control.

## Materials and methods

### Bacterial strains and growth conditions

The bacterial strains constructed and used are listed in Table 1 (Vaccine candidates tested) and Additional file 1 (List of strains and plasmids used). All strains were stored at −80 °C in cryoprotector media: skim milk (Scharlau) or TSBY-7% DMSO (tryptic soy broth (Scharlau) supplemented with 0.5% yeast extract (Pronadisa, Condalab) and dimethyl sulfoxide (VWR)).

**Table 1:** **Vaccine candidates tested**

Strain	Characteristics	Relevant phenotype	Reference
H38Δ*wbkF*	H38 carrying an internal deletion in *wbkF* gene	Rough LPS	[32]
H38Δ*wbkF*Δ*wadB*	H38 carrying an internal deletion in *wbkF* and *wadB* genes	Rough Truncated core LPS	This work
H38Δ*wbkF*Δ*wadC*	H38 carrying an internal deletion in *wbkF* and *wadC* genes	Rough Truncated core LPS	This work

For mutant construction and in vitro characterization, bacteria were routinely grown in tryptic soy broth (TSB, Scharlab S.L, Barcelona, Spain) or TSB with bacteriological agar (TSA; Agar Condalab, Madrid, Spain) at 37 °C for 3–4 days in air. For strain selection in some steps of mutagenesis (final mutants lack antibiotic resistance markers; see below), tryptic soy agar was supplemented with kanamycin (Km) at 50 μg/mL, nalidixic acid (Nal) at 25 μg/mL, and/or 5% sucrose.

To prepare the inoculum for studies in mice and rams, vaccine strains were grown on Blood Agar Base plates (BAB2, Oxoid, UK) and incubated in air to obtain 24–48 h fresh cultures. *B. ovis* PA and *B. ovis* PA::Tn7Km^R^ [43] challenge strains (Additional file 1), were grown on BAB2 with 5% fetal bovine serum (Gibco) plates (BAB2S) in a 10% CO_2_ atmosphere.

### DNA sequence analysis

Searches for DNA and protein homologies were carried out using the Kyoto Encyclopedia of Genes and Genomes (KEGG) database, and protein sequence alignments were performed with the EMBL-EBI Clustal Omega tool.

### Construction of H38Δ*wbkF*Δ*wadB* and H38Δ*wbkF*Δ*wadC* mutants

The plasmids used are described in Additional file 1. In-frame deletion mutants H38Δ*wbkF*Δ*wadB* and H38Δ*wbkF*Δ*wadC* were respectively constructed using the mutator plasmids pYRI-2 [44], which contains the *wadB* deletion allele, and pRCI-26 [45], which contains the *wadC* deletion allele. The corresponding plasmid was introduced into H38Δ*wbkF* by conjugation with *Escherichia coli* S17-1 λpir [46]; the first recombination was selected by nalidixic and kanamycin resistance, and the double recombination by nalidixic and sucrose resistance and kanamycin sensitivity [44, 45]. The deletion of *wadB* was confirmed by PCR with oligonucleotides *wadB-*F1 and *wadB-*R4 [44], which yielded 570-bp or 1011-bp fragments for the mutant and the H38Δ*wbkF* sibling revertant strain, respectively. For the mutation in *wad*C, the primers used were *wadC*-F1 and *wadC*-R4 [45], and the amplified fragments were 929 bp and 1805 bp in the double mutant and the H38Δ*wbkF* sibling revertant strain, respectively. Mutations were complemented by introducing plasmids p*wadB* [44] and p*wadC* [45] into the respective mutants by mating with *E. coli* S17-1 λpir followed by selection of the conjugants on TSA-Nal-Km plates.

### Phenotypical characterization of mutants

Mutants were characterized by standard *Brucella* typing procedures [47]. In brief, strains were examined for CO_2_ requirement and susceptibility to *Brucella* phages (Tb (Tbilisi), Wb (Weybridge), Iz (Izatnagar) and R/C), urease and oxidase, and agglutination with anti-A and anti-M sera. The S/R colony morphology was determined by the crystal violet dye exclusion test. To assess growth, overnight cultures obtained in TSB were adjusted to an OD_600_ of 0.1, 200 µL inoculated in triplicate in TSB-containing Bioscreen C plates, and growth was monitored at 37 °C with continuous shaking by measuring absorbance at 420–580 nm every 30 min in a Bioscreen C (Lab Systems) apparatus.

LPS from H38, H38Δ*wbkF*, H38Δ*wbkF*Δ*wadB,* and H38Δ*wbkF*Δ*wadC* strains was extracted for phenotypical characterization following the proteinase-K sodium dodecyl sulfate (SDS) protocol [48, 49] with slight modifications. In brief, each strain was grown overnight in a total of 80 mL of TSB and inactivated the following day using 0.5% phenol. After inactivation, cells were washed twice with saline and weighed before being suspended by sonication in 2% SDS-60 mM Tris–HCl buffer (pH 6.8) at a concentration of 0.5 g (wet weight) of bacteria per 10 mL of buffer. Suspensions were heated at 100 °C for 10 min and cooled to 55 °C. Proteinase K (60 µL at 2.5 mg/mL in HCl–Tris) was added, and digestion was carried out for 3 h at 55 °C, followed by an overnight incubation at 20 °C. The LPS present in the supernatant was then precipitated by adding 3 volumes of methanol containing 1% sodium acetate-saturated methanol and incubating at −20 °C for 3 h. After 12 h, the precipitate was collected by centrifugation at 5000 *g* for 15 min at 4 °C and resuspended by sonication in 10 mL of distilled water. Following a second methanol precipitation and centrifugation, the pellets were resuspended by sonication in 2–3 mL of 60 mM HCl–Tris (pH 6.8) and incubated at 37 °C. Samples were then treated with DNase and RNase (60 µL at 0.5 mg/mL in HCl–Tris) at 37 °C for 30 min. A second proteinase K treatment was performed under the same conditions (3 h, 55 °C), followed by a third methanol precipitation. The final LPS pellets were dissolved in 1 mL of distilled water and stored at −20 °C until analysis. LPS samples were mixed 1:1 (v:v) with sample buffer 2× (Bio-Rad), heated at 100 °C for 10 min, and analyzed in 12% Bis–Tris Precast gel (Bio-Rad) or 18% polyacrylamide gels (37.5:1 acrylamide:methylene-bisacrylamide). For western blot, gels were electrotransferred onto nitrocellulose blotting membrane (Amersham, Merck, Germany) of 0.45 µm pore size and analyzed with a serum from a rabbit immunized with anti-*B. abortus* 2308Δ*per* [50] and monoclonal antibody A68-24G12/A08, which recognizes core epitopes [51]. Due to inconsistent extraction yields among samples and a uniform resuspension volume (1 mL), LPS concentrations may vary between preparations. Thus, western blot results should be interpreted qualitatively rather than quantitatively.

### Virulence and protection studies in mice

All procedures were in accordance with the current European (Directive 2010/63/EU) and Spanish (RD 53/2013 and 1386–2018) legislations on the protection of animals used for scientific purposes and approved by the Ethical Committee of each institution and local Governments. Five-week-old female BALB/C mice (ENVIGO, Harlan) were kept in groups of five animals in cages at CITA BSL-3 facilities (ES502970012005) with water and food ad libitum.

For virulence assessment, groups of five mice were inoculated intraperitoneally with 1 × 10^8^ colony-forming units (CFU)/mouse of H38Δ*wbkF*, H38Δ*wbkF*Δ*wadB* or H38Δ*wbkF*Δ*wadC* or 1 × 10^4^ CFU per mouse of the H38 parental strain, and CFU in spleen measured 1, 3, and 5 weeks after inoculation. Results are expressed as the mean log_10_ ± standard deviation (SD) [52].

For protection assessment, groups of five mice were vaccinated by the subcutaneous (SC) route with 1 × 10^8^ CFU per mouse of each candidate (H38Δ*wbkF*, H38Δ*wbkF*Δ*wadB* or H38Δ*wbkF*Δ*wadC*), 1 × 10^5^ CFU per mouse of Rev1 (protective vaccine control) or phosphate-buffered saline (non-immunized control). After 4 weeks, mice were challenged intraperitoneally with 1 × 10^6^ CFU per mouse of the *B. ovis* virulent strain tagged with kanamycin (Km) resistance, *B. ovis* PA:Tn7Km^R^ [43]. Two weeks later, mice were euthanized, and the mean log_10_ CFU per spleen ± SD of the challenge strain was determined. For this, spleen samples were plated on both BAB2S (which supports the growth of both vaccine and challenge strains), and BAB2S supplemented with 5% kanamycin (BAB2S-Km), on which only the *B. ovis* PA:Tn7Km^R^ challenge strain grows. CFU counts from BAB2S-Km were used to quantify the challenge bacteria and calculate units of protection, while the numerical difference between CFU counts on BAB2S and BAB2S-Km was used to estimate residual vaccine (not shown). The genetic stability of *wbkF* mutation of the spleen isolates was confirmed by PCR (Additional file 2) and crystal violet exclusion test at several points during the infection process. Statistical comparisons were made by the one-way analysis of variance (ANOVA) with Dunnett’s multiple comparison or Fisher’s protected least significant differences (PLSD) post-hoc tests.

### Protection of H38Δ*wbkF* in rams

#### Ram vaccination and challenge

All experiments were performed in compliance with the current legislation on the protection of animals used for scientific purposes and approved by the Ethical Committee and local Government.

A total of 35 brucellosis-free Rasa Aragonesa rams aged 4 months were randomly allotted in three pens that were maintained separated throughout the experiment with water and food provided ad libitum.

According to retrospectively assessed CFU counts, rams from the first group (*n* = 12) were vaccinated conjunctivally (CJ) with 1.5 × 10^10^ CFU of H38Δ*wbkF*. The other two groups served as controls: one was CJ-vaccinated with 1 × 10^9^ CFU of the commercial Rev1 vaccine (Ocurev ^®^, *n* = 11), while the other remained unvaccinated (*n* = 12).

Eight months (32 weeks) after vaccination (both vaccine strains are cleared at this time), all rams were challenged with the virulent *B. ovis* PA strain, a well-characterized field isolate from Pyrénées Atlantiques, originally obtained at Institut National de la Recherche Agronomique (INRA, France) and preserved at Centro de Investigación y Tecnología Agroalimentaria de Aragón (CITA) (the strain virulence had been previously confirmed in mice). Based on our own experience and previous reports [26, 32, 33], the challenge inoculum was adjusted spectrophotometrically to achieve a target dose of 1–3 × 10^9^ CFU. Retrospective CFU counts indicated that each animal ultimately received 4 × 10^9^ CFU of *B. ovis* PA, administered via both the conjunctival (30 µL) and preputial (30 µL) routes. Then, rams were examined weekly for clinical signs and symptoms of infection (genital lesions, fever, apathy, or anorexia).

#### Bacteriological studies

Eight weeks after the challenge, rams were euthanized and thoroughly necropsied. Testes and epididymides were examined for specific lesions. Portions of spleen and epididymides, whole seminal vesicles, and cranial (submaxillary, parotid and retropharyngeal), iliac, scrotal, crural, and prescapular lymph nodes of each animal were taken for bacteriological examination. All samples were processed and cultured on the same day they were obtained. In brief, samples were degreased, externally sterilized by dipping into absolute ethanol and gentle flaming, and placed in a sterile bag. Then, each sample was cut into small pieces and homogenized in the minimum possible amount of buffered saline solution using a Stomacher^®^ (London, UK). One milliliter of each homogenate was seeded onto duplicate plates of CITA medium [53] and incubated at 37 °C in a 10% CO_2_ atmosphere. After 5–7 days, suspicious colonies were examined using standard procedures [47], including anti-A/anti-M agglutination and crystal violet staining. Bacterial DNA was extracted from pure cultures using the Speedtools Tissue DNA Extraction Kit (Biotools, Madrid, Spain), and *Brucella* species were identified by Bruce-ladder multiplex PCR [54]. These techniques allow rapid differentiation between smooth (Rev1 vaccine) and rough (*B. ovis* PA) colonies, as well as between *B. melitensis* H38Δ*wbkF* and *B. ovis* PA isolates. One animal was considered infected when at least one *B. ovis* PA CFU was isolated from any of the eight samples seeded. Infection levels for each organ were categorized as follows: (1) 1–5 CFU, (2) 6–25 CFU, (3) 26–125 CFU, (4) 126–250 CFU, and (5) > 250 CFU per plate. Organs showing an infection level ≥ 3 were considered severely infected. Statistical comparisons of the number of *B. ovis* infected animals were made using the chi-square test, and numbers of infected organs per animal were compared using STEPBOOT MULTTEST (5.0, SAS Institute Inc. Copyright^©^).

#### Serological studies

Blood samples were taken by venipuncture before starting the experiment, weekly for the first 4 weeks after vaccination and challenge, and fortnightly during the rest of the experiment. The serological response against S *Brucella* was analyzed using RBT and CFT [42] and an S-LPS iELISA [55], and the serological responses against *B. ovis* using the WOAH-recommended AGID [42], and an HS iELISA (*B. ovis* PA HS) [32]. Optimal serum dilution and cutoffs (i.e., the [100 × OD_sample_/OD_positive control_] value providing 100% diagnostic specificity) were determined previously for *B. melitensis* (1/50 dilution and 50% OD cutoff) and *B. ovis* (1/100 dilution and 40% OD cutoff) iELISAs using panels of gold-standard sera from brucellosis-free sheep, and *B. melitensis* or *B. ovis* culture-positive sheep.

## Results

### Construction of potential DIVA vaccine candidates based on LPS core defects

**Figure 1:**
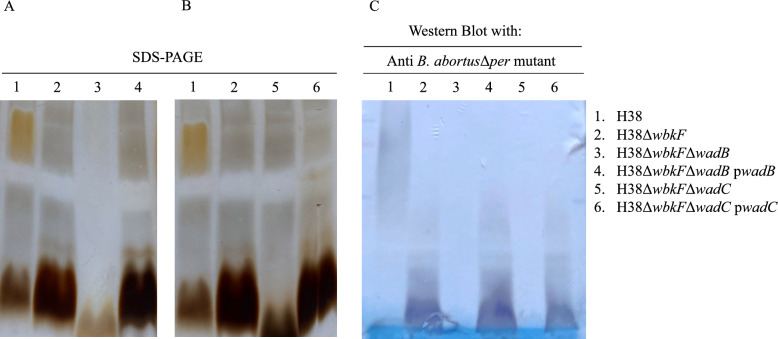
**H38Δ*wbkF*Δ*wadB* and H38Δ*wbkF*Δ*wadC* present a defective LPS core oligosaccharide**. **A** SDS–PAGE electrophoresis and silver staining of SDS–proteinase K LPS extracts. **B** Western blot analysis of SDS–proteinase K. **C** LPS extracts with anti-R-*Brucella* polyclonal serum.

We first confirmed that the *wadB* and *wadC* orthologous genes from *B. abortus* in *B. melitensis* H38 are highly conserved (data not shown), a step necessary to construct nonpolar mutants H38Δ*wbkF*Δ*wadB* and H38Δ*wbkF*Δ*wadC* using the available *B. abortus* genetic tools (Additional file 1) [44, 45]. Then, we examined the corresponding LPS phenotypes. In comparison with H38Δ*wbkF*, the LPS core region of H38Δ*wbkF*Δ*wadB* and H38Δ*wbkF*Δ*wadC* showed the predicted smaller molecular weight indicative of the lack of some sugars in the core oligosaccharide lateral branch (Figure 1A). Moreover, western blot analysis using monoclonal antibody A68/24G12/A08-specific for *Brucella* LPS core epitope (not shown) and polyclonal anti-R-*Brucella* serum (Figure 1B) showed that the defect in the core epitope of H38Δ*wbkF*Δ*wadB* and H38Δ*wbkF*Δ*wadC* abrogated the reactivity of anti-R-LPS antibodies. Complementation of H38Δ*wbkF*Δ*wadB* and H38Δ*wbkF*Δ*wadC* with *wadB* and *wadC,* respectively, restored the electrophoretic migration pattern and the antibody reactivity of parental H38Δ*wbkF* (Figure 1B). Conventional phenotyping showed the anticipated results for the parental S H38 strain (Additional file 3), while for H38Δ*wbkF*, H38Δ*wbkF*Δ*wadB* and H38Δ*wbkF*Δ*wadC* the results in the crystal violet exclusion test (not shown), R/C phage susceptibility, and agglutination with acriflavine or anti-A/M antisera were consistent with those of R *Brucella*e [47]*.* H38Δ*wbkF*Δ*wadB* and H38Δ*wbkF*Δ*wadC* were identical to H38Δ*wbkF* in growth rates in rich media, and the three mutants showed delayed growth compared with the parental H38 strain (Additional file 4).

### The *wadB* and *wadC* deletions in H38Δ*wbkF* lead to over-attenuation and lack of protection in the mouse model

**Figure 2:**
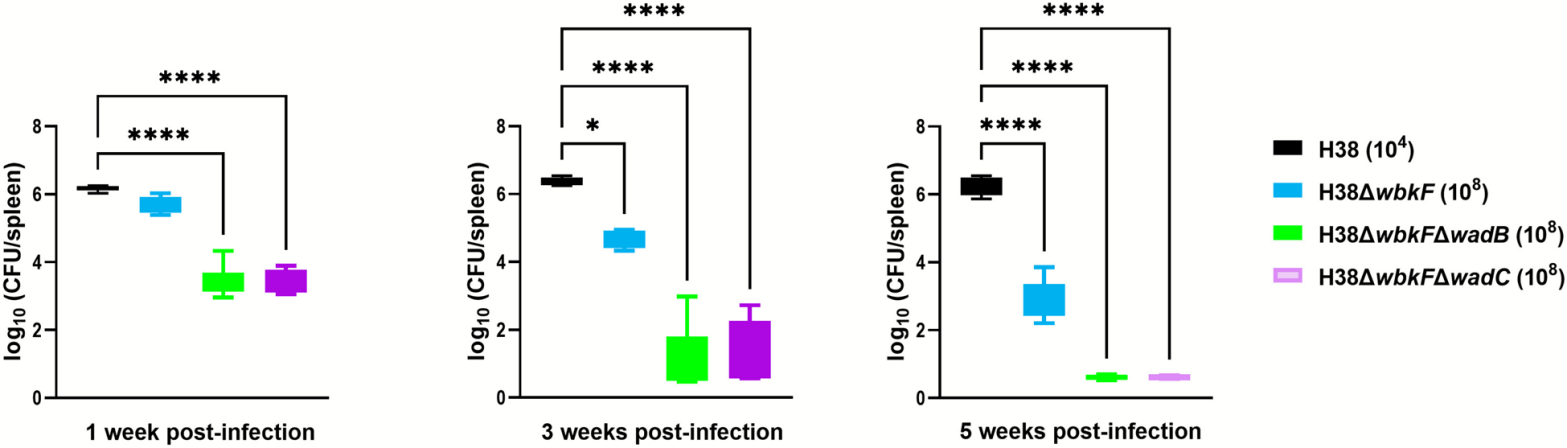
**Multiplication of the vaccine candidates in mice**. BALB/C mice were inoculated with the indicated doses and CFU per spleen determined at the indicated intervals (*p < 0.05, **p < 0.01, ***p < 0.001, ****p < 0.0001). One-way ANOVA followed by Dunnett’s multiple-comparison test.

The virulence of the potential DIVA-double mutants H38Δ*wbkF*Δ*wadB* and H38Δ*wbkF*Δ*wadC* was evaluated in mice in comparison with wild-type H38 and the parental H38Δ*wbkF* (Figure 2). For all mutants, infection levels peaked in week 1 and then declined progressively. By the third week after infection, spleen CFU had significantly decreased in all vaccine candidate groups, whereas the wild-type H38 strain maintained the high bacterial loads characteristic of virulent S *Brucella*e. Notably, both double mutants were significantly more attenuated than H38Δ*wbkF*, which behaved consistently with previous findings [32]. This higher attenuation of the double mutants resulted in a nonstatistically significant protection against *B. ovis* (Table 2). These results made us abandon the modification of H38Δ*wbkF* LPS core as a potential DIVA strategy and focus on CJ administration to reduce the H38Δ*wbkF*-induced serological interference.

**Table 2:** **Protection against *B. ovis* PA-km**^**R**^** in mice**

Vaccine (CFU dose)	*B. ovis* PA-Km^R^ (mean ± SD of log_10_ CFU per spleen)	Units of protection ^a^
H38Δ*wbkF* (10^8^)	3.42 ± 0.62 ^b, c^	2.87
H38Δ*wbkF*Δ*wadB* (10^8^)	4.69 ± 2.82	1.61
H38Δ*wbkF*Δ*wadC* (10^8^)	5.64 ± 0.74	0.66
Rev1 (10^5^)	2.45 ± 1.35 ^b^	3.84
Non-immunized control	6.29 ± 0.38	

^a^Units of protection: average log_10_ CFU challenge strain in the spleens of non-immunized control minus average log_10_ CFU of the challenge strain in the spleens of vaccinated mice.

^b^Significant difference (*P* < 0.005) *vs* non-immunized control.

^c^No significant differences (*p* > 0.05) compared with the Rev1-vaccinated control.

### CJ administration of H38Δ*wbkF* and Rev1 similarly protect rams against *B. ovis*

**Figure 3:**
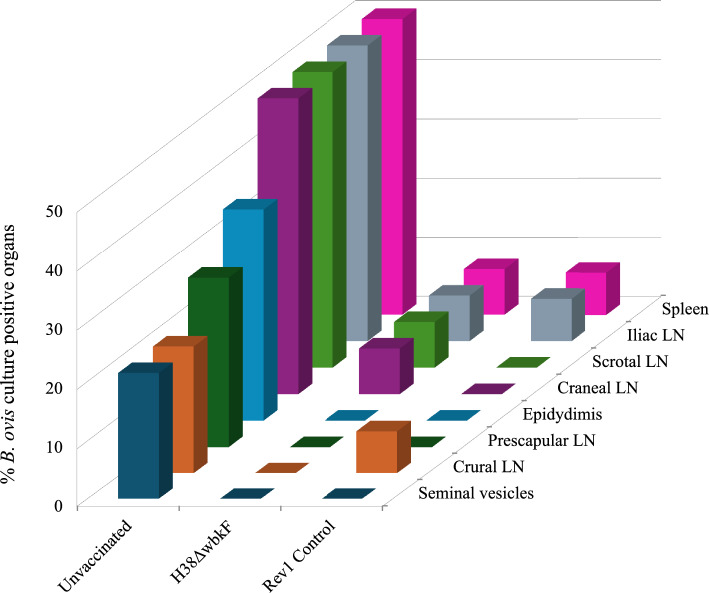
**Percentages of *B. ovis* culture-positive organs in each experimental group of rams**. LN, lymph nodes.

Relevant results related to vaccine protection are summarized in Table 3 and Figure 3. Demonstrative of the severity of the challenge, colonization by *B. ovis* was extensive in multiple lymph nodes, spleen, and/or reproductive organs, and severe testicular lesions were observed in four animals of the unvaccinated group. The proportion of infected animals (83.3%) and samples (44.8%) in this group was significantly higher (*p* < 0.001) than in the Rev1 control group, in which we detected only one infected animal (9.1%) and only in three organs (3.4%). These results enabled robust statistical comparisons, highlighting the suitability of the experiment for proper evaluations. The level of protection conferred by H38Δ*wbkF* (just one infected animal) was comparable to that achieved with Rev1, and the percentage of infected organs was similarly low (4.2%). The proportion of samples showing infection levels ≥ 3 (i.e., severely infected; see “Materials and methods”) was significantly higher in the nonvaccinated animals (21%) compared with the H38Δ*wbkF* (2%) and Rev1 (0%) groups. The most frequently infected tissues were the epididymis and the iliac, scrotal, and cranial lymph nodes, consistent with the challenge routes used (preputial and conjunctival). Overall, the outcomes paralleled those observed after SC immunization [32].

**Table 3:** **Protective efficacy of conjunctival vaccination in rams against a *B. ovis* PA challenge**

Vaccine	No. infected animals/total (%)^a^	No. infected organs/total (%)^b^
H38Δ*wbkF*	1/12 (8.3)^c, d^	4/96 (4.2)^c, d^
Rev1	1/11 (9.1)^c^	3/88 (3.4)^c^
Unvaccinated	10/12 (83.3)	43/96 (44.8)

^**a**^Statistical comparison by chi-square test (with Fisher–Yates correction when required).

^**b**^Statistical comparisons by STEPBOOT MULTTEST (SAS).

^c^High significant difference (*p* < 0.001) versus unvaccinated control. ^d^No significant (*p* > 0.05) versus Rev1-vaccinated group.

### CJ administration of H38Δ*wbkF* minimized serological interference in rams

**Figure 4:**
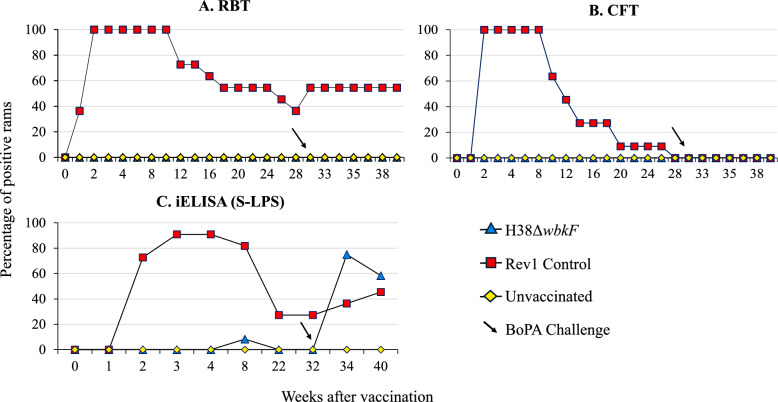
**Evolution of antibody response in S-LPS-based tests used for *B. melitensis* diagnostic after conjunctival vaccination (week 0) and challenge (week 32)**

The evolution of the response in RBT and CFT is shown in Figure 4 (panels A and B). As expected, all animals in the Rev1-vaccinated group developed positive serological reactions within 2 weeks, which gradually declined after week 10 and became negative in CFT at week 28, but persisted positive in RBT until the end of the experiment. By contrast, those vaccinated with H38Δ*wbkF* remained seronegative throughout the assay. The S-LPS-iELISA (Figure 4, panel C) revealed only a transient positive reaction in one animal of the H38Δ*wbkF* group, whereas most of the Rev1 controls remained positive, which is consistent with previous reports. Following the *B. ovis* challenge, a significant proportion of the vaccinated animals developed antibodies detectable by this S-LPS-iELISA (Figure 4), as expected because of the cross-reactivity of the LPS core epitopes.

**Figure 5:**
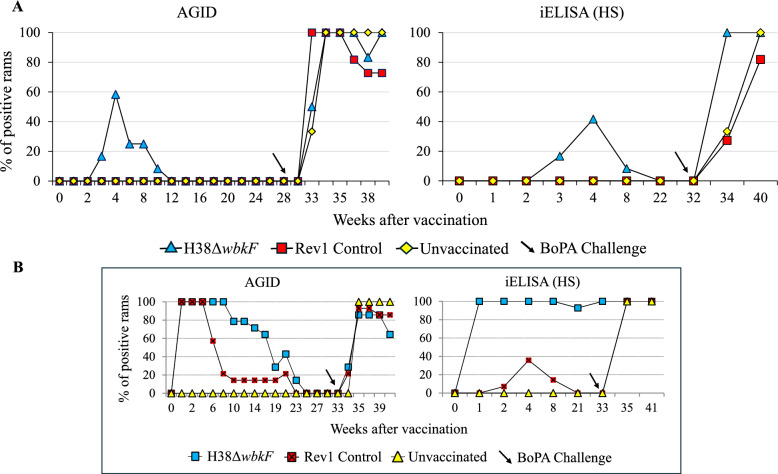
**Evolution of antibody response in tests used for *B. ovis* diagnosis**. **A** Percentage of seropositive rams after conjunctival vaccination (week 0) and challenge (week 32) in agar gel immunodiffusion (AGID) and indirect ELISA (iELISA) tests using *B. ovis* hot saline (HS) extract. **B** Serological response of rams vaccinated subcutaneously in the same tests (data extracted from Muñoz et al. [32].

The evolution of the antibody response in tests using *B. ovis* HS extracts is shown in Figure 5 (panels A and B). Both unvaccinated and Rev1 CJ-vaccinated controls remained negative in *B. ovis* tests (AGID and HS-iELISA) until challenge. By contrast, the H38Δ*wbkF*-vaccinated group showed transient positive responses in a moderate proportion of animals (42% and 58%, by week 4, in AGID and HS-iELISA, respectively). These responses rapidly declined and completely disappeared by 8–10 weeks in both tests. These results contrast with those of the previous SC vaccination trial, where positive reactions persisted throughout the experiment (Figure 5, panel B). As expected, all animals were positive in *B. ovis* serological tests after challenge.

## Discussion

Rev1 vaccination is pivotal for the control and eradication of ovine brucellosis caused by *B. melitensis* [10, 40] and simultaneously prevents the spread of *B. ovis* [15–17]. However, in *B. melitensis*-free regions, where the use of Rev1 is not permitted, there is a pressing need for a specific *B. ovis* vaccine. An optimal vaccine should not only avoid disrupting *B. melitensis* serosurveillance but also minimize the interference of *B. ovis* testing programs.

Positive tagging with green fluorescent protein (GFP) has been explored as a DIVA strategy for brucellosis vaccines S19 and Rev1 [33, 56, 57]. The strategy uses constructs carrying the *gfp* gene in a neutral site of the genome, but as the GFP expressed does not trigger an antibody response strong enough, the *gfp* tagged vaccine is supplemented with recombinant GFP produced in *Escherichia coli* and purified by affinity chromatography [57]. In a recent study with a limited number (*n* = 4) of animals, sheep were inoculated with Rev1Δ*wzm::gfp* plus free recombinant GFP (the study did not include rams inoculated with GFP alone) [34], and sera were analyzed using a GFP-iELISA and a commercial *Brucella* R-ELISA as part of a diagnostic DIVA strategy. The Rev1Δ*wzm::gfp*-free GFP combination induced long-lasting antibodies against both *B. ovis* antigens and GFP, as expected, but protection in these animals was not assessed. Assuming that the Rev1Δ*wzm*::*gfp* is not affected by the genetic manipulations necessary to insert *gfp* in the genome that could reduce the protection obtained in the same work with Rev1Δ*wzm*, about 30% of the vaccinated animals would contain the challenge strain in genital organs [33]. Also, a proportion of Rev1Δ*wzm*-vaccinated animals tested positive in RBT and CFT, as observed before with both spontaneous and transposon *wzm* mutants for periods of up to 6–7 months after vaccination [33–37, 58–60].Thus, Rev1Δ*wzm::gfp*-vaccinated sheep could simultaneously test positive for S-LPS, R-LPS, and GFP and, since a proportion may become infected despite vaccination [33], a positive GFP would not totally discriminate *B. melitensis* or *B. ovis* infected and noninfected sheep. These uncertainties illustrate the problems of positively tagged brucellosis vaccines and the confusing epidemiological picture they can generate when they carry immunogenic O-PS epitopes.

In brucellosis, a classical approach to address the problems created by postvaccinal antibodies in eradication programs has been the removal of diagnostic epitopes, and it led to the development of R vaccines totally devoid of O-PS [28, 61]. Although R vaccines (including *wzm* mutants) are unsatisfactory against brucellosis of cattle and small ruminants caused by S *Brucella* spp. because of the excessive attenuation caused by the O-PS deficiency [61–63], sheep infections by *B. ovis* are a different scenario on account of both its R antigenic structure and, when compared with *B. melitensis*, lower virulence. These differences probably explain why R H38Δ*wbkF*, which is devoid of the O-PS but keeps an intact core oligosaccharide, protects rams against *B. ovis* via SC vaccination without interfering with routine *B. melitensis* tests [32]. Nevertheless, as the best *B. ovis* serological tests use HS extracts that are rich in R-LPS and outer membrane proteins [4, 7–9, 38, 64], SC H38Δ*wbkF* triggers positive responses in both HS AGID and iELISA [32]. Thus, following the hypothesis that deletion of diagnostic epitopes could mitigate the interference, we deleted core sugars of the lateral branch characteristic of the *Brucella* LPS core by disrupting two of the three glycosyltransferases involved (WadB, WadC, and WadD). This five-sugar branch hinders recognition by the TLR4-MD2 receptor system so that mutants in *wadB*, *wadC* and *wadD* are attenuated by the subsequently enhanced Th1 immune response and display vaccine potential in the mouse model, as shown for *B. abortus wadB*, *wadD* and *wadC* mutants, *B. suis* biovar 2 *wadB* and *wadD* mutants and *B. ovis wadC* and *wadB* mutants [32, 44, 45, 65–68]. However, despite promising results in mice, Bov:CAΔ*wadB* failed to protect rams against *B. ovis* [32], possibly because of an excessive attenuation related to a prompt recognition by innate immunity that leads to nonsustained cellular immunity. In this experiment, the sera of most rams vaccinated with Bov::CAΔ*wadB* reacted in AGID with homologous *wadB* HS, but not in the AGID with wild-type HS, suggesting that modifying the R-LPS epitopes could mitigate the serological interference of *B. ovis* R vaccines constructed in a protective background. Consequently, we investigated whether core defects could reduce the serological interference of H38Δ*wbkF* without affecting its protective efficacy. However, mutants H38Δ*wbkF*Δ*wadB* and H38Δ*wbkF*Δ*wadC* failed to confer protection in mice, in all likelihood because they exhibited accelerated clearance related to a ready recognition that impairs the development of an effective cellular immune response. Additional studies assessing cellular and humoral immune response markers could provide insights into the mechanisms underlying the lack of protective efficacy observed for the *wadB* and *wadC* mutants in R background.

Our second strategy to reduce H38Δ*wbkF*-induced serological interference was based on the use of the CJ route for vaccine administration. It is well established that, while Rev1 SC vaccination promotes systemic dissemination and induces persistent antibody responses, CJ administration limits dissemination and antibody duration, aligning better with test-and-slaughter eradication strategies. Both field and experimental evidence support the use of CJ route for Rev1 [18, 40, 41, 69], which generates robust immunity not only in the oropharyngeal mucosae, the main portal of natural *Brucella* infection, but also in distant areas. Our findings extend these observations to CJ-administered H38Δ*wbkF* because it conferred optimal protection against a *B. ovis* challenge administered through both the conjunctival and preputial routes. The proportions of infected versus uninfected animals in the H38Δ*wbkF* and Rev1-vaccinated groups and in the nonvaccinated control group not only validate the present experiment by ensuring reliable statistical comparisons but also confirm those of the previous SC vaccination trial [32], given the similar infection rates across both studies.

Consistent with the data obtained after SC vaccination, CJ vaccination with H38Δ*wbkF* did not interfere in either the RBT or CFT before or after the *B. ovis* challenge, thus confirming its compatibility with *B. melitensis* surveillance programs. However, although cross-reactivity in the S-LPS iELISA was notably reduced compared with that seen after SC administration [32], it increased after *B. ovis* challenge. These observations are explained by the fact that the LPS core epitopes shared by the S and R-LPS of *Brucella* are not accessible to antibodies in the S *Brucella* cells used as antigens in RBT and CFT but become accessible in ELISAs upon adsorption of the S-LPS to plastic matrixes [6]. Thus, in *B. melitensis*-free regions affected by *B. ovis,* or areas where R vaccines are used, RBT, rather than S-LPS iELISA, is the most reliable and cost-effective option for *B. melitensis* surveillance.

With regard to the H38Δ*wbkF* interference in *B. ovis* serodiagnosis, CJ vaccination markedly reduced the intensity and persistence of cross-reacting antibodies in both AGID and iELISA with wild-type *B. ovis* HS antigens. While this represents a clear practical improvement, these results need to be complemented with field observations, particularly in highly infected areas where repeated exposure to *B. ovis* field strains might reactivate or prolong antibody response in H38Δ*wbkF*-vaccinated sheep, as observed with other brucellosis vaccines in endemic settings [6]. Overall, conjunctival H38Δ*wbkF* vaccination is a promising approach to support *B. ovis* eradication programs in regions free of *B. melitensis*.

## Supplementary Information

**Additional file 1. List of strains and plasmids used.**

**Additional file 2. PCR amplicons generated with primers F1-R4 distinguish the WT H38 (1796 bp) from the H38Δ*wbkF* mutant (953 bp), confirming stable maintenance of the engineered locus.**

**Additional file 3. Bacteriological characterization of *Brucella* H38 and its derived mutants according to standard phenotyping procedures **[47]**.**

**Additional file 4. Growth defects of different *B. melitensis* H38-derived mutants in TSB. Results are shown as growth curves of each strain in TSB media. **At each time point, values represent the mean ± SD of one representative experiment performed in technical triplicates. SDs are displayed as dotted lines above and below the main curve. The experiment was repeated three times with similar results.

## Data Availability

No datasets were generated or analyzed during the current study.
