# Supplementary material for: Conjunctival administration of H38ΔwbkF rough vaccine as an effective strategy to protect against Brucella ovis infection while minimizing serological interference
Source: Vet Res. 2026 Mar 8;57:50. doi: 10.1186/s13567-025-01693-8 (PMC13081411; doi:10.1186/s13567-025-01693-8)
Supplement: Supplementary file 1 — Additional file 1. List of strains and plasmids used. [file 13567_2025_1693_MOESM1_ESM.docx]

| **Additional File 1. List of strains and plasmids used** | | | |
| --- | --- | --- | --- |
| ***Brucella* strains** | | **References** | |
| H38 | *B. melitensis* WT smooth virulent strain | | [58] |
| *B. ovis* PA | Challenge strain used in *B. ovis* vaccine studies in rams; virulent | | [23] |
| *B. ovis* PA::Tn7Km^R^ | *B. ovis* PA with mini Tn7 transposon (pUC18R6KTminiTn7T-Km). Challenge strain in mice studies. | | [69] |
| ***Escherichia coli*** | | **References** | |
| *E. coli* S17-1 λpir | Mating strain with plasmid RP4 inserted into the chromosome | | [46] |
| **Plasmids** | | **References** | |
| pRCI-26 | *Bam*HI-*Xba*I fragment from pRCLI-23 (carrying the *wadC* deletion allele) cloned into the corresponding sites of pJQK | | [45] |
| p*wadC* | attL1- attL2 fragment of pDONR201- BMEI0509 (carrying the complete *wadC* gene) cloned into the attR1- attR2 sites of pRH001 | | [45] |
| pYRI-2 | *Bam*HI-*Xba*I fragment from pYRI-1 (containing the *wadB* deletion allele) cloned into the corresponding sites of pJQK | | [44] |
| p*wadB* | attL1-attL2 fragment of pYRI-3 (containing the complete *wadB* gene) cloned into the attR1-attR2 sites of pRH001 | | [44] |
