# Supplementary figures and images for: Conjunctival administration of H38ΔwbkF rough vaccine as an effective strategy to protect against Brucella ovis infection while minimizing serological interference
Source: Vet Res. 2026 Mar 8;57:50. doi: 10.1186/s13567-025-01693-8 (PMC13081411; doi:10.1186/s13567-025-01693-8)

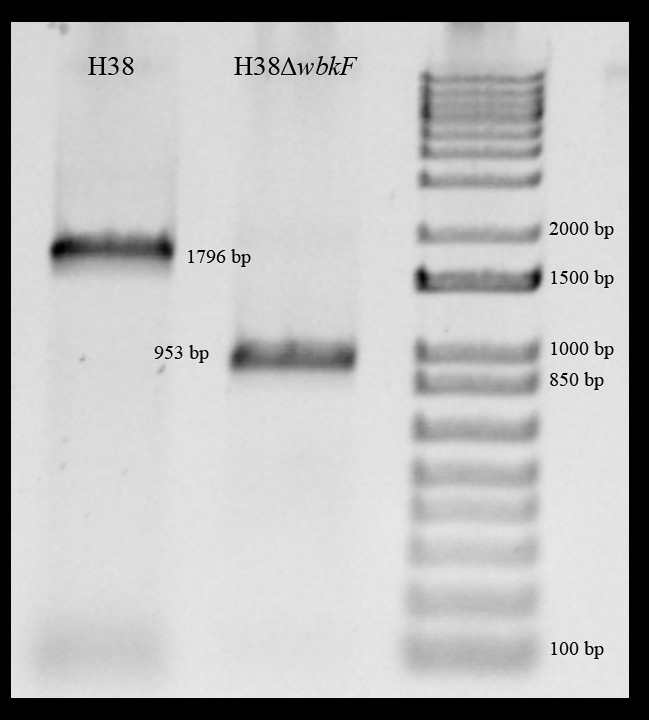

Supplement: Supplementary file 2 — Additional file 2. PCR amplicons generated with primers F1-R4 distinguish the WT H38 (1796 bp) from the H38ΔwbkF mutant (953 bp), confirming stable maintenance of the engineered locus. [file 13567_2025_1693_MOESM2_ESM.png]

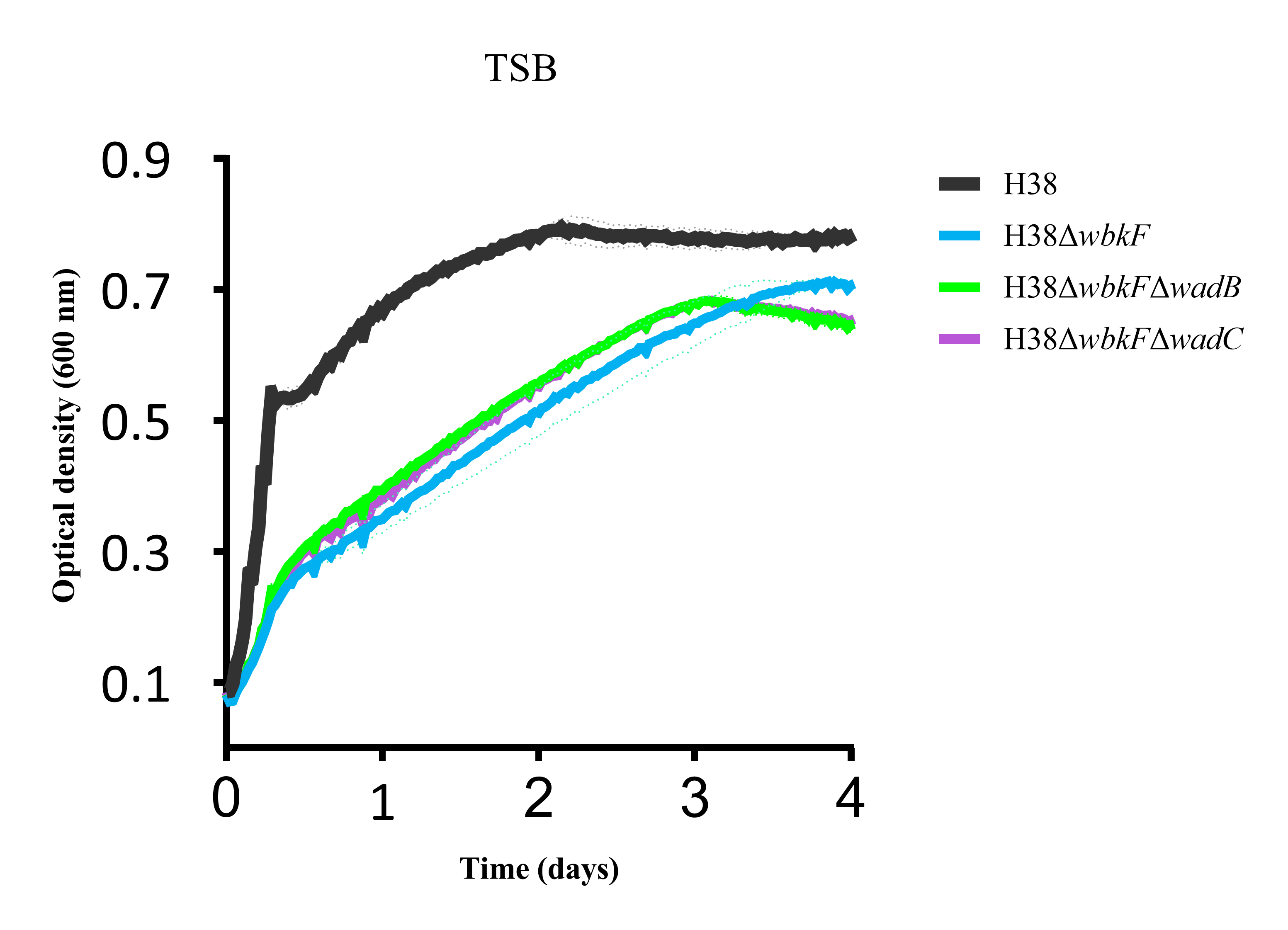

Supplement: Supplementary file 4 — Additional file 4. Growth defects of different B. melitensis H38-derived mutants in TSB. Results are shown as growth curves of each strain in TSB media. At each time point, values represent the mean ± SD of one representative experiment performed in technical triplicates. SDs are displayed as dotted lines above and below the main curve. The experiment was repeated three times with similar results. [file 13567_2025_1693_MOESM4_ESM.tif]
