# Supplementary material for: Conjunctival administration of H38ΔwbkF rough vaccine as an effective strategy to protect against Brucella ovis infection while minimizing serological interference
Source: Vet Res. 2026 Mar 8;57:50. doi: 10.1186/s13567-025-01693-8 (PMC13081411; doi:10.1186/s13567-025-01693-8)
Supplement: Supplementary file 3 — Additional file 3. Bacteriological characterization of Brucella H38 and its derived mutants according to standard phenotyping procedures [47]. [file 13567_2025_1693_MOESM3_ESM.docx]

| **Additional File 3. Bacteriological characterization of *Brucella* H38 and its derived mutants according to standard phenotyping procedures [47].** | | | | | | | | | |
| --- | --- | --- | --- | --- | --- | --- | --- | --- | --- |
|  | **Lysis by phages^1^** | | | | **CO_2_** | **Urease activity** | **Acriflavine agglutination** | **Serum agglutination** | |
|  | **Tb** | **Wb** | **Iz** | **R/C** |  |  |  | **A** | **M** |
| **H38** | **-** | **-** | **+** | **-** | **-** | **+** | **-** | **-** | **+** |
| **H38Δ*wbkF*** | **-** | **-** | **-** | **+** | **-** | **+** | **+** | **-** | **-** |
| **H38Δ*wbkF*Δ*wadB*** | **-** | **-** | **-** | **+** | **-** | **+** | **+** | **-** | **-** |
| **H38Δ*wbkF*Δ*wadC*** | **-** | **-** | **-** | **+** | **-** | **+** | **+** | **-** | **-** |
| ^1^Results using routine test dilution (RTD). Phages: Tbilisi (Tb), Weybridge (Wb), Izatnagar (Iz) and R/C | | | | | | | | | |
